# Protect the Brain When Treating the Heart: Feasibility of 2.5D U-Net for Real-Time Gaseous Microemboli Detection

**DOI:** 10.3390/bioengineering13091017

**Published:** 2026-09-01

**Authors:** Andrea Angino, Ken Trotti, Diego Ulisse Pizzagalli, Rolf Krause, Tiziano Torre, Stefanos Demertzis

**Affiliations:** 1 Center for Computational Medicine in Cardiology (CCMC), Università della Svizzera Italiana (USI), 6900 Lugano, Switzerland; rolf.krause@usi.ch; 2 Faculty of Mathematics and Computer Science, UniDistance Suisse, 3900 Brig, Switzerland; 3 Applied Mathematics and Computational Science (AMCS), Computer, Electrical and Mathematical Sciences and Engineering (CEMSE) Division, King Abdullah University of Science and Technology (KAUST), Thuwal 23955-6900, Saudi Arabia; ken.trotti@kaust.edu.sa; 4 Faculty of Biomedical Sciences, Università della Svizzera Italiana (USI), 6900 Lugano, Switzerland; stefanos.demertzis@eoc.ch; 5 Theodor Kocher Institute, Faculty of Medicine, University of Bern, 3012 Bern, Switzerland; 6 Institute of Digital Technologies for Personalised Healthcare (MeDiTech), Department of Innovative Technologies, University of Applied Sciences and Arts of Southern Switzerland (SUPSI), 6962 Lugano-Viganello, Switzerland; 7 Cardiac Surgery Department, Cardiocentro Ticino Institute, Ente Ospedaliero Cantonale (EOC), 6900 Lugano, Switzerland; tiziano.torre@eoc.ch; 8 Faculty of Medicine, University of Bern, 3012 Bern, Switzerland

**Keywords:** computer vision, biomedicine, echocardiography, gaseous microemboli, U-Net, real-time segmentation, cardiac surgery

## Abstract

Gaseous microemboli (GME) represent a common complication of cardiac structural interventions across both surgical and transcatheter approaches. Intraoperative transesophageal echocardiography (TEE) represents a convenient methodology to monitor and visualize the presence of circulating GME. However, their detection and quantification are far from trivial due to operator-dependent view, high velocity, and objects with similar structure in the background. Here, we propose a feasibility study based on a 2.5D U-Net architecture to detect GME in space-time connected data. We applied and tested such an architecture on a pilot dataset of eight TEE recordings (60 fps, 600×800 pixels) from eight different patients undergoing cardiac surgery, resulting in improved detection of moving GMEs against the background with respect to classical spot detection algorithms and 2D U-Net, yet retaining real-time execution speed with respect to more complex deep-learning architectures. Under leave-one-patient-out cross-validation, the selected model achieved strong detection performance under a three-pixel radius-tolerant grace-zone evaluation, with a precision of 92.55% and recall of 80.54%, corresponding to radius-tolerant Intersection over Union (IoU) and Dice coefficients of 73.95% and 84.13%, respectively. Complementarily, strict pixel-based segmentation metrics were also computed, yielding an IoU of 41.74% and a Dice coefficient of 57.98%. The selected model achieved an average inference time of 0.12 s per batch on the tested hardware. To assess specificity on unseen data, we additionally evaluated the model on an external GME-negative TEE dataset, where it produced predominantly empty or near-empty masks, indicating a low rate of spurious detections. These results support the technical feasibility of real-time GME segmentation, although broader clinical validation on larger multi-patient, multicenter datasets is still required.

## 1. Introduction

Cardiac structural interventions (surgical and/or transcatheter) are associated with significant neurological risks due to the inadvertent introduction of emboli—solid or gaseous—into the bloodstream. Gaseous microemboli (GME), composed of environmental air, are especially concerning as they can travel through the circulatory system with a slow reabsorption rate, potentially lodging in cerebral vessels and leading to silent infarctions and cognitive deficits [1]. Neurological complications arising from GME have been documented extensively, ranging from transient impairments to long-term cognitive decline in patients following cardiac procedures [2]. Existing solutions, such as transcranial Doppler ultrasound and diffusion-weighted MRI, provide a means of postoperative emboli detection, yet they lack the immediacy necessary to facilitate real-time intervention during surgery, limiting their effectiveness in mitigating intraoperative neurological risks [3]. Providing real-time feedback within the surgical workflow can therefore support the operating team by offering timely confidence estimates and improving intraoperative decision-making [4].

Cardiac echocardiography stands as an effective procedure that can be easily integrated into clinical practice to detect GMEs in cardiac chambers. Among the imaging modalities, trans-esophageal echocardiography (TEE) is particularly convenient for intraoperative monitoring. However, reliable software for automatic GME detection and quantification is still lacking. This gap is largely explained by the small size of emboli, their irregular and variable appearance, and their strong similarity to surrounding tissue and common echocardiographic artifacts, which makes purely appearance-based detection unreliable.

The contribution of this work is threefold: first, we describe a dedicated annotation workflow for GME detection in intraoperative echocardiographic videos. Second, we adapt a lightweight 2.5D U-Net to exploit short-term temporal information while preserving real-time feasibility. Third, we integrate the segmentation output into a prototype monitoring pipeline that provides frame-wise GME area estimates. Hence, we provide a technical feasibility study on a pilot dataset, that could set the basis for a more complete clinical validation and a support to the doctors during image segmentation to build the dataset.

## 2. Related Work

Learning-based segmentation provides a natural way to address these challenges. State-of-the-art architectures such as Fully Convolutional Networks (FCNs) [5] and Mask R-CNN [6] have been successfully applied to medical image segmentation, while U-Net remains a widely adopted reference model due to its ability to preserve fine spatial details through skip connections [7]. Beyond static segmentation, recent work has shown that U-Net-based models can also segment motile targets in grayscale images with limited resolution [8]. However, when the input consists of video or temporally structured imaging data, relying on single-frame appearance alone can be limiting, especially when the target objects are small, rapidly moving, and visually similar to background artifacts. In such cases, short-term temporal information can provide discriminative cues that are not available from individual frames. This motivates the use of 2.5D approaches, in which a short stack of adjacent frames or slices is provided as input, offering a practical compromise between purely 2D models and computationally heavier 3D architectures.

In the specific case of GME, appearance alone is often insufficient as emboli may be indistinguishable from static speckle patterns and transient artifacts in single frames, while their rapid motion across consecutive frames provides a key cue for discrimination. Building on this evidence, we propose a real-time detection system based on a lightweight 2.5D U-Net architecture for GME segmentation in echocardiographic video. The model processes short sequences of consecutive frames to introduce limited temporal context while remaining computationally feasible for online deployment under real-time intraoperative constraints.

## 3. Dataset Construction

Segmenting GME requires meticulous, time-intensive work by trained specialists capable of distinguishing microemboli from surrounding cardiac structures.

Segmentation in this context presents unique challenges: while it may initially seem straightforward to delineate hyperechogenic regions within each frame, the task is complicated by the fact that cardiac muscle and emboli often share similar grayscale intensities on echocardiographic evaluation. This similarity creates ambiguity, especially near edges, where motion is the key indicator distinguishing rapidly moving emboli from the slower-moving or stationary cardiac tissue. Manual analysis typically requires moving through video frames to observe these subtle differences in movement, as GMEs are characterized by rapid positional changes compared to the relatively stable cardiac background.

**Figure 1:**
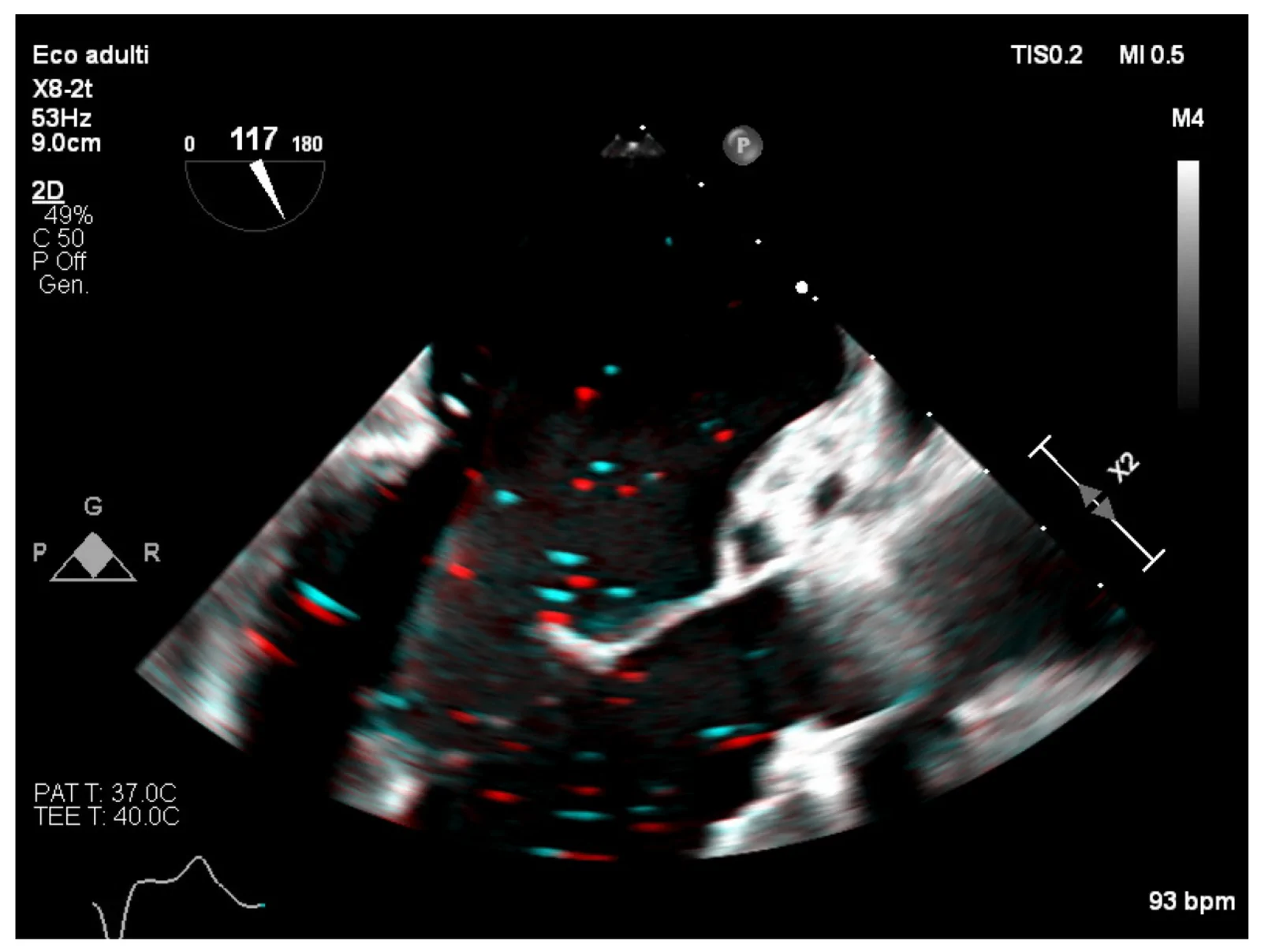
Color-coded overlay of two consecutive frames: the initial frame is depicted in red, while the subsequent frame appears in turquoise. Accordingly, red-only regions correspond to structures at their position in the initial frame, whereas turquoise-only regions correspond to their position in the subsequent frame. Regions overlapping at the same position in both frames appear white, corresponding to the stable cardiac background. The distinct red/turquoise displacement highlights the dynamic movement of GMEs.

Figure 1 presents two overlapping subsequent frames, with the first shown in red and the second in turquoise. This color-coded overlay highlights motion cues, enabling the precise identification of emboli against the stationary cardiac background.

For this study, we developed a custom annotation tool tailored to meet the specific requirements of GME segmentation in echocardiographic frames. This tool provides intuitive navigation through video frames, allowing expert annotators to segment emboli using two selection modes: a manual outline tool for precise boundary tracing and an automatic region selection tool that identifies regions based on color variation. Although the automatic mode expedites the segmentation process, it can occasionally lack precision, making manual corrections necessary. The tool also includes user-friendly features, such as an “undo” function to reverse recent actions, a “cancel” option for discarding wrongly selected regions, and an auto-save feature to ensure that all changes are securely stored. The annotation tool and supporting software resources are openly available in the AirCatch GitHub v1.0.0 repository [9]. The repository includes installation instructions, dependency information, and a Quick Start example to facilitate the use of the software.

Annotations were generated with a custom tool for manual annotation, and clinically reviewed under the supervision of the cardiac surgery specialists among the authors (T.T. and S.D.). Ambiguous regions were assessed by inspecting consecutive frames, since motion relative to the cardiac background was the main criterion for identifying GME. A post hoc independent annotation reproducibility analysis was subsequently performed on a randomly selected subset of the dataset and is reported in Section 3.

Although frame subdivision increases the number of localized training samples, the dataset remains limited in terms of independent clinical acquisitions.

Because of the limited number of source videos, the present results should be interpreted as pilot within-dataset feasibility results rather than as proof of patient-independent generalization, which will require larger and more diverse multi-patient datasets.

### Post Hoc Inter-Observer Agreement Analysis

To provide a quantitative assessment of annotation reproducibility, 30 frames were randomly selected from the annotated dataset and independently re-annotated by a clinician in training who had not previously seen either the dataset or the original annotations. The re-annotation was performed with access to adjacent frames but without access to the reference masks. The original consensus annotations are denoted by (A), while the independent re-annotations are denoted by (B).

Individual GMEs were extracted from each binary mask using connected-component analysis. The centroid of each connected component was defined as the mean (x)- and (y)-coordinates of all pixels belonging to that object. Objects in (A) and (B) were matched one-to-one according to the Euclidean distance between their centroids, using a maximum matching distance of 5 pixels. The 5-pixel threshold was chosen to allow small centroid shifts due to contour differences while avoiding matches between spatially distinct GMEs.

**Figure 2:**
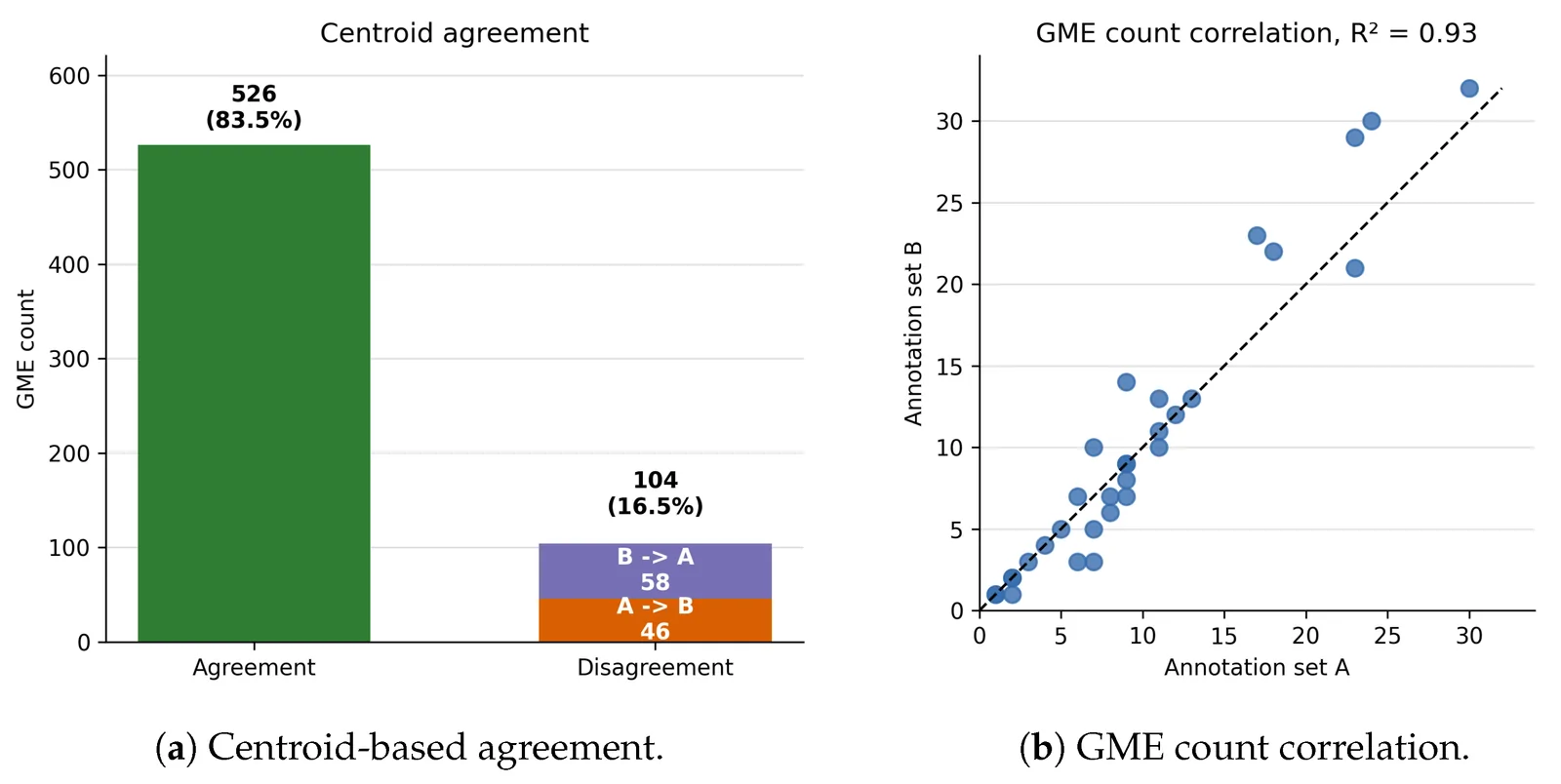
Post hoc inter-observer analysis on 30 randomly selected frames. Panel (**a**) shows object-level agreement between the original consensus annotations (A) and the independent re-annotations (B), based on one-to-one centroid matching with a maximum centroid distance of 5 pixels. Green indicates matched centroids, while orange and purple indicate unmatched annotations from A to B and from B to A, respectively. Panel (**b**) shows the frame-wise correspondence between the numbers of GMEs identified in (A) and (B). Observed correlation $R^{2}=0.93$.

Across the 30 re-annotated frames, 526 GME objects were matched between the two annotation sets, while 46 objects in (A) and 58 objects in (B) had no corresponding match. In addition, the frame-wise numbers of annotated GMEs showed strong correspondence between the two annotation sets, with $R^{2}=0.93$, as shown in Figure 2.

This analysis indicates reproducibility in identifying and spatially localizing individual GME objects. It should nevertheless be distinguished from pixel-wise contour reproducibility. Qualitative inspection of matched annotations showed that independent observers could identify the same GME while assigning different boundaries. Such differences are particularly relevant for small objects represented at limited spatial resolution, since TEE provides a two-dimensional representation of a moving three-dimensional GME. Consequently, small variations in boundary placement may substantially affect strict pixel-wise overlap even when object identity and localization are concordant. These observations provide additional empirical support for considering the proposed radius-tolerant metrics introduced in Section 4.2 as complementary measures.

## 4. Neural Network Structure

**Figure 3:**
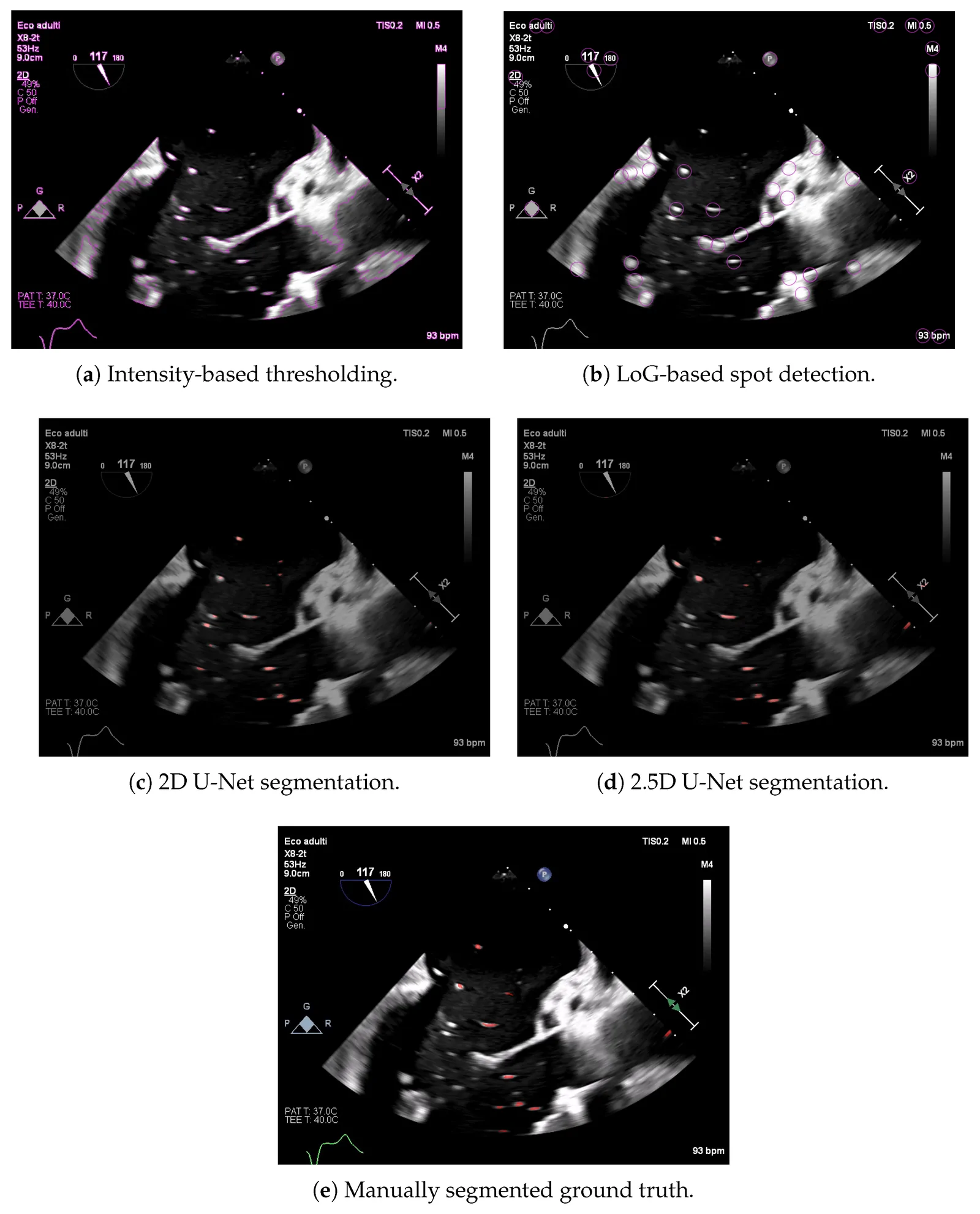
Representative qualitative comparison of segmentation results obtained using classical image-processing methods (**a**,**b**), deep learning-based models (**c**,**d**), and manually segmented ground truth (**e**). In panels (**a**–**d**), colored overlays indicate the regions segmented as GMEs by each method, superimposed on the original echocardiographic frame. Panel (**e**) shows the manually annotated ground-truth GME regions using the same overlay convention. Color-coded markings highlight the detected or segmented regions on the original echocardiographic frame: purple markings indicate the outputs of the classical methods in panels (**a**,**b**), while red overlays indicate segmented GME regions in panels (**c**–**e**).

To provide a comparative baseline, we first evaluated classical segmentation approaches using the open-source Fiji/TrackMate v7.13.0 [10] software (Thresholding and Laplacian of Gaussian (LoG) filtering strategies). The top-left and top-right panels in Figure 3 correspond to the segmentation outputs obtained with these methods. As can be seen, both approaches capture not only the GMEs but also portions of the surrounding muscular tissue, resulting in over-segmentation. The LoG method shows improved sensitivity to GMEs but still fails to suppress responses from cardiac structures with similar intensity and texture characteristics.

These limitations of traditional image processing approaches highlighted the need for a learning-based strategy capable of capturing more complex spatial and contextual features, motivating our decision to adopt a deep learning framework.

A 2D U-Net architecture, which is widely recognized for its effectiveness in biomedical image segmentation [7], struggles to effectively distinguish between GME and the surrounding cardiac boundaries due to the limited size of our dataset and the intrinsic complexity of differentiating microemboli from surrounding tissue structures.

Considering the importance of motion cues in differentiating emboli from background tissue, an observation made during dataset construction, the main improvement in model architecture was achieved by transitioning to a 2.5D U-Net [11]. Unlike a 3D U-Net, which processes a full volumetric space-time block of data, and hence is not suitable for real-time processing, a 2.5D U-Net partially incorporates the temporal dimension by including a short sequence of frames along the time axis. This allows the network to capture essential temporal information while remaining computationally efficient for real-time applications. As shown in Figure 3c,d, comparing with the manually segmented reference in Figure 3e, the 2D model often misclassifies background resembling GMEs and overestimates the true GMEs area, whereas the 2.5D model excludes the background.

The choice of a U-Net architecture stems from its well-documented success in biomedical imaging tasks due to its encoder–decoder structure [12], which enables detailed segmentation by leveraging contextual information. The U-Net’s skip connections allow the model to retain spatial information lost during downsampling, crucial for accurately segmenting GME boundaries against a complex background.

All patients undergoing cardiac surgery are equipped with a direct invasive arterial line, a central venous catheter, and a bladder catheter. Under conditions of general anesthesia and orotracheal intubation, a trans-esophageal ultrasound probe is positioned, which allows continuous monitoring of cardiac contractility, the functionality of the heart valves, and the result of the operation performed. The video frames were acquired by the echocardiographic machine (Philips Epiq) during the weaning phases from the Cardiopulmonary Bypass (CPB) when any bubbles present inside the cardiac cavities are pushed forward by the resumption of pulmonary ventilation. To enable real-time processing and sharing, an HDTV video capture USB 3.0 device was used to connect the Philips Epiq to a laptop, mirroring the echocardiographic images while capturing high-definition video for NN analysis. The NN processes batches of 10 frames, introducing a delay of 0.16 s for image loading and batch formation during which the previous batch is processed, and a batch inference time of 0.12 s, ensuring near real-time visualization with a bounded overall delay of approximately 0.28 s. This setup allows doctors to perform their assessments as if the AI were not present, relying solely on the live echocardiographic feed. However, they can also refer to the laptop screen to visualize quantitative bubble estimates and the regions identified by the neural network as potential GMEs, providing an additional layer of insight.

The binary segmentation procedure, described in Algorithm 1, takes a raw grayscale frame as input and applies a pretrained U-Net to produce a binary mask. The input frame is first divided into four quadrants, which are processed in batches by the network. The resulting per-pixel probabilities are reassembled into a full-frame probability map and thresholded to obtain the final binary segmentation.
**Algorithm 1:** 2.5D U-Net segmentation with frame length history lh
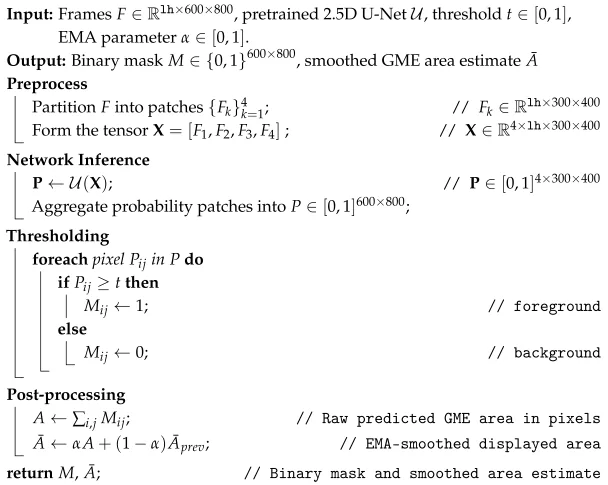


### 4.1. Hardware Requirements

For practical real-time deployment of the proposed parallel processing pipeline, we recommend a system equipped with an 8 GB or larger CUDA-enabled GPU and an 8-core processor. This configuration should be regarded as a practical deployment recommendation rather than an experimentally established minimum hardware threshold. This setup ensures energy and resource efficiency and can be physically located in the operating room near the echocardiography machine. The machine requires multiple cores to distribute the workload across several processing pipelines. One pipeline is dedicated to the NN’s inference, another is responsible for computing the estimate by processing the segmented data through an exponential moving average, effectively prioritizing recent changes while retaining long-term trends for accuracy, and a third for generating the real-time output, which results from combining the previous contributions into a coherent visualization. This division of tasks ensures seamless integration and uninterrupted real-time operation, even in resource-constrained environments.

### 4.2. Segmentation Performance Metrics

To comprehensively evaluate segmentation performance, we consider three complementary metrics: the Frequency Bias Index (FBI), the Intersection over Union (IoU), and the Dice coefficient. Each captures different aspects of segmentation quality, allowing for a nuanced assessment of model behavior in detecting GME regions.

Throughout this section, we use the following notation:

- TPs (true positives): number of GME pixels correctly predicted as GME,
- FPs (false positives): number of background pixels incorrectly predicted as GME,
- FNs (false negatives): number of GME pixels missed by the model,
- TNs (true negatives): number of background pixels correctly predicted as background.

#### 4.2.1. Frequency Bias Index

The Frequency Bias Index (FBI) is used in this work as a primary calibration metric for estimating the overall GME burden. Since the clinical objective is to quantify the total embolic area visible in the echocardiographic stream, FBI provides a direct measure of whether the predicted GME extent is systematically under- or over-estimated. It is commonly used in forecasting to measure the tendency of a model to over- or under-predict positive events. In our context, it is defined as the ratio between the number of pixels predicted as GME and the total number of ground-truth GME pixels, rescaled to a percentage for visualization purposes:

(1) $$\mathrm{FBI}=\frac{\mathrm{TP}+\mathrm{FP}}{\mathrm{TP}+\mathrm{FN}}\times 100=\left(1+\frac{\mathrm{FP}-\mathrm{FN}}{\mathrm{TP}+\mathrm{FN}}\right)\times 100.$$

A value close to 100% indicates that the predicted segmentation has approximately the same area as the ground-truth GME, regardless of its spatial alignment. For this reason, FBI should be interpreted as a quantitative area-estimation metric rather than as a measure of spatial localization accuracy. Spatial agreement between prediction and annotation is therefore evaluated separately through IoU and Dice metrics. The right-hand side of Equation (1) highlights how the FBI reflects the net discrepancy between predicted and true GME regions: if FN>FP, the model underestimates the GME extent and the FBI falls below 100%. Conversely, if FP>FN, the model overestimates it and the FBI exceeds 100%.

This property makes the FBI particularly suitable for our application, where capturing the full extent of the GME is critical, as a slight overestimation (Type I error dominant, FP>FN), which may lead to unnecessary deairing procedures, is more acceptable than underestimation (Type II error dominant, FN>FP), which could result in failing to intervene on patients at risk due to high GME levels. Thus, a model with an FBI slightly above 100% is preferable to one below 100%, as it reflects a conservative yet balanced estimate of the GME area.

#### 4.2.2. Intersection over Union (IoU)

The Intersection over Union (IoU), or Jaccard index, quantifies the overlap between predicted and true GME regions in terms of pixel counts. It is defined as

(2) $$\mathrm{IoU}\;=\;\frac{\mathrm{TP}}{\mathrm{TP}+\mathrm{FP}+\mathrm{FN}}\times 100\%.$$

An IoU of 100% means perfect agreement, while 0% means no overlap. Because every FP and FN reduces the score equally, IoU is a balanced measure of segmentation quality.

#### 4.2.3. Dice Coefficient

The Dice coefficient (also called the $F_{1}$-score for segmentation) emphasizes the harmonic mean of precision and recall. In terms of pixel counts:

(3) $$\mathrm{Dice}\;=\;\frac{2\;\mathrm{TP}}{2\;\mathrm{TP}+\mathrm{FP}+\mathrm{FN}}\times 100\%.$$

Dice ranges from 0% (no overlap) to 100% (perfect overlap). By giving double weight to TP, Dice is more sensitive to small structures and less punishing of isolated FP or FN than IoU.

Consequently, model selection was based on the combined interpretation of FBI, IoU, and Dice, with particular emphasis on FBI due to the quantitative embolic-load estimation objective of the study.

#### 4.2.4. Radius-Tolerant Grace-Zone Evaluation

The main objective of this study is reliable GME detection and embolic-load estimation rather than exact contour agreement at single-pixel resolution. Therefore, in addition to strict pixel-wise segmentation metrics described above, we introduce a radius-tolerant grace-zone evaluation that tolerates small contour errors around annotated GME masks, focusing on GME detection. This is appropriate for our application because, in this formulation, predictions falling close to the annotated GME boundaries are treated as acceptable detections rather than hard segmentation errors.

The radius-tolerant metrics provide a complementary assessment of GME detection in the presence of small boundary ambiguities.

Let Y⊆Ω denote the ground-truth mask, let $\hat{Y}\subseteq \Omega$ denote the predicted mask, and let $D_{r}$ be the discrete disk of radius *r* pixels. To account for boundary uncertainty, we define radius-tolerant neighborhoods around both the annotation and the prediction as

$$Y_{r}=Y⊕D_{r},\;{\hat{Y}}_{r}=\hat{Y}⊕D_{r}.$$

Under the following formulation, the radius-tolerant confusion terms are defined as

$${FP}_{r}^{\mathrm{eff}}=|\hat{Y}\setminus Y_{r}|,\;FN_{r}^{\mathrm{eff}}=\left|Y\setminus {\hat{Y}}_{r}\right|,$$

$$TP_{r}^{\mathrm{eff}}=\frac{1}{2}\left(TP_{r}^{\mathrm{pred}}+TP_{r}^{\mathrm{gt}}\right).\;TN_{r}^{\mathrm{eff}}=\left|\Omega \setminus \left(Y_{r}\cup \hat{Y}\right)\right|,$$

where

$$TP_{r}^{\mathrm{pred}}=|\hat{Y}\cap Y_{r}|,\;TP_{r}^{\mathrm{gt}}=\left|Y\cap {\hat{Y}}_{r}\right|.$$

IoU, Dice, precision, recall, and $F_{1}$ are then computed from these radius-tolerant counts and denoted with subscript *r*. In this way, small boundary mismatches are penalized less severely, while clear false detections in safe background and missed GME cores remain penalized.

**Figure 4:**
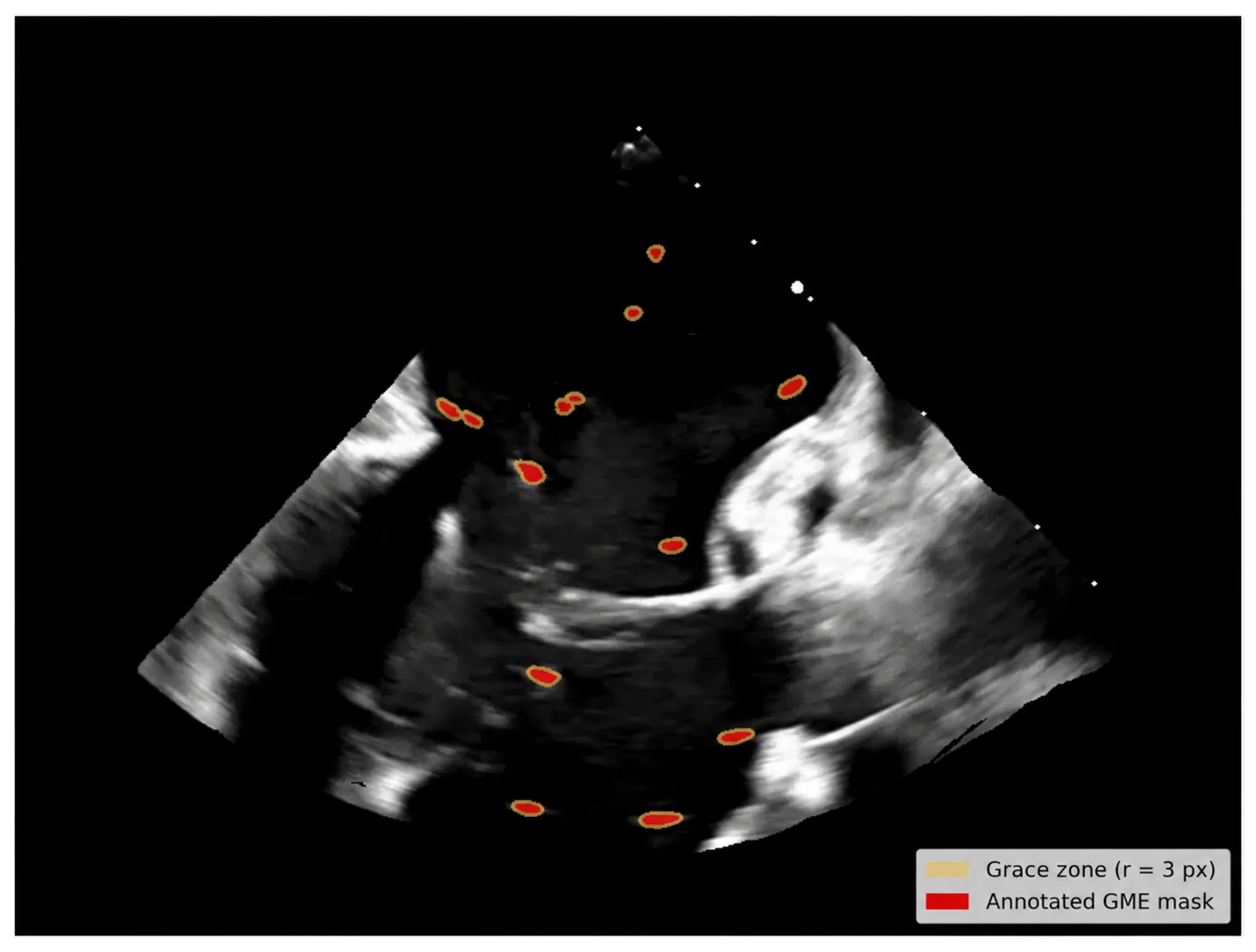
Illustration of the radius-tolerant grace-zone evaluation with a grace-zone radius of r=3 px. Red regions represent the annotated GME mask, while the yellow bands correspond to the tolerance regions generated by the structuring element $D_{r}$. Predicted foreground pixels falling inside these regions are treated as partially correct detections and therefore contribute to the effective true-positive term instead of being counted as strict false positives. The same tolerance principle is applied symmetrically to the prediction mask through ${\hat{Y}}_{r}$.

An example of this strategy is illustrated in Figure 4 with r=3, where the yellow regions correspond to the tolerance bands generated by $D_{r}$ around the annotated GMEs. Predicted foreground pixels falling inside these regions are treated as partially correct detections and therefore contribute to the effective true-positive term rather than being counted as strict false positives. The same reasoning is applied symmetrically to the prediction mask through ${\hat{Y}}_{r}$.

Tolerance-based evaluation is common in boundary-aware and medical-image segmentation, where small localization errors are accepted when predicted and reference contours fall within a prescribed distance [13,14,15]. Following this idea, we define a radius-tolerant mask evaluation in which predicted positives within a radius-*r* neighborhood of the ground-truth GMEs mask are counted as valid detections, while unpredicted pixels inside this neighborhood are treated as ambiguous rather than rewarded as true negatives.

The sensitivity of the resulting metrics to the choice of the radius *r* is evaluated empirically in Section 5.3.

## 5. Numerical Results

This section presents all experimental results. In Section 5.1, we investigate the effect of input length, specifically, the number of frames provided to the 2.5D U-Net. Section 5.2 presents a parameter study used to finalize the model configuration. For both Section 5.1 and Section 5.2, the dataset was split into 80% training and 20% testing sets for model selection and hyperparameter analysis. Section 5.3 reports the performance of the selected model evaluated using leave-one-patient-out cross-validation.

All experiments in Section 5.1, Section 5.2 and Section 5.3 were conducted under the same training conditions, as detailed below. All models were trained for 50 epochs with a batch size of 10, using binary cross-entropy loss and Adam optimizer. The learning rate was set to $\mathrm{lr}=10^{-3}$ for the first 24 epochs and then reduced to $\mathrm{lr}=10^{-4}$ thereafter, using the default momentum values of $\beta_{1}=0.9$ and $\beta_{2}=0.999$. Results are averaged over three runs with different random initializations. Experiments were conducted on a Raider A18 HX A9W laptop (Micro-Star International Co., Ltd., New Taipei City, Taiwan; AMD Ryzen 9 9955HX3D CPU (Santa Clara, CA, USA), 64 GB RAM, NVIDIA RTX 5080 GPU with 16 GB VRAM (Santa Clara, CA, USA)). Section 5.4 evaluates the models on the GME-negative SR-TEE dataset [16] to assess spurious detections.

Finally, Section 5.5 presents a qualitative real-time validation of the final system integrated into the online inference pipeline.

### 5.1. Impact of Model History Length

In the first experiment, we evaluate the impact of input sequence length on the performance of a 2.5D U-Net, specifically whether more than two consecutive frames offer any benefit. Due to delay constraints associated with longer histories, we limit the maximum history to three frames.

**Figure 5:**
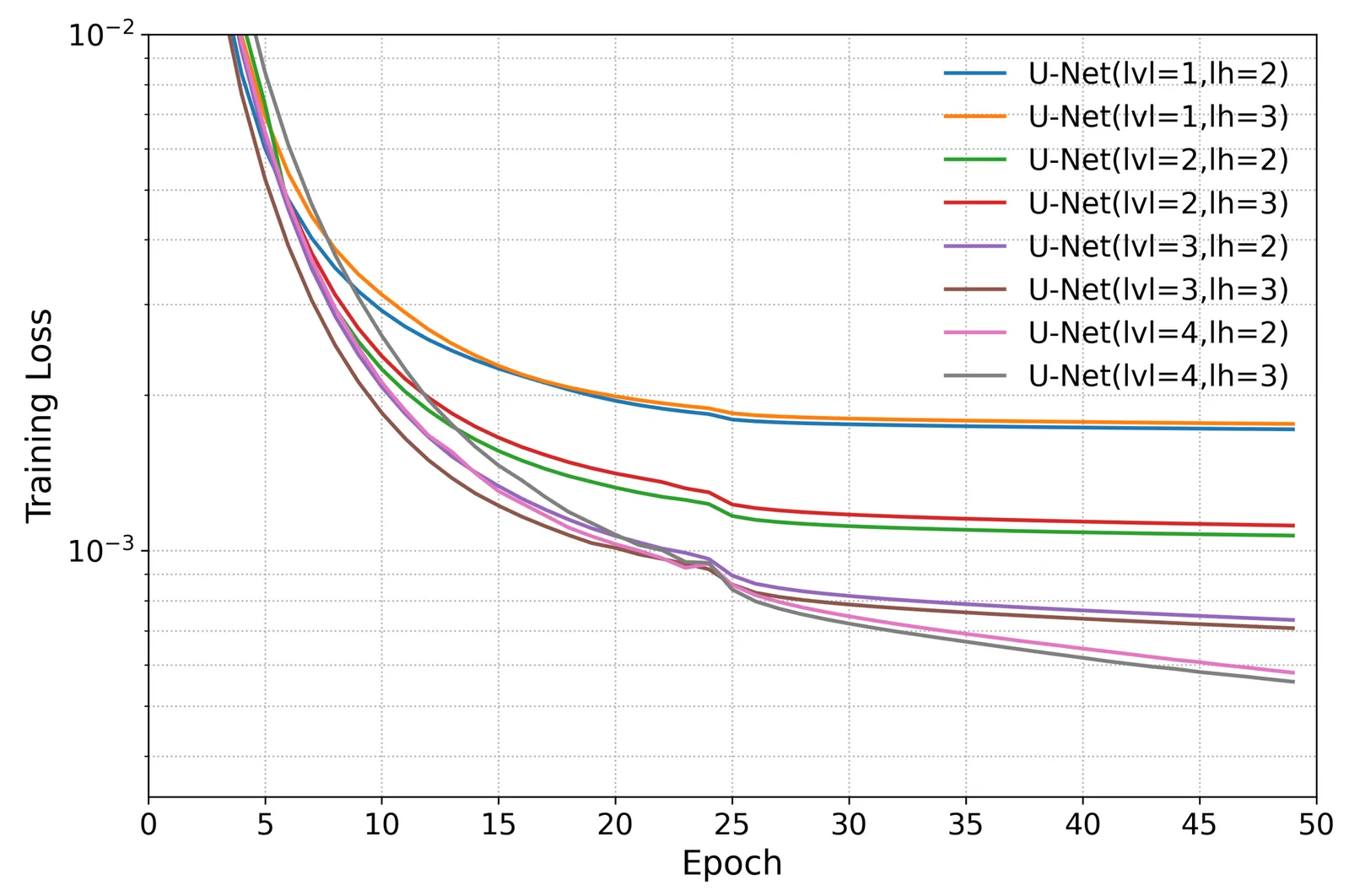
Training loss over 50 epochs for 2.5D U-Net models with 8 base channels, varying the number of down-sampling levels (lvl ∈{1,2,3,4}) and the input history length (lh ∈{2,3}). All models were trained using binary cross-entropy loss and the Adam optimizer with a batch size of 10.

Figure 5 presents the training loss curves as a function of epochs for a 2.5D U-Net for different parameters. Specifically, the base number of feature map channels is set to 8 as we vary the number of downsampling levels lvl ∈{1,2,3,4} and the input history length (lh ∈{2,3}).

As expected, increasing the number of downsampling levels leads to a faster decrease in training loss. Moreover, for shallow models (lvl ∈{1,2}), using two frames (lh =2) yields a small reduction in loss compared with those using lh =3, while for deeper models (lvl ∈{3,4}), three frames (lh =3) perform slightly better than the lh=2 counterparts. However, the difference at epoch 50 remains below 4% and a longer history incurs extra computation and latency due to the need to wait for an additional future frame. At 60 frames/s, each additional frame corresponds to approximately 16.7 ms of acquisition delay before inference can be performed. Since the performance improvement from lh =3 remained below 4%, lh =2 was retained to preserve lower latency under real-time constraints. Based on this trade-off and the limitation given by the delay, we consider two input frames sufficient to distinguish moving foreground from static background, and accordingly fix lh =2 in all subsequent experiments.

Training was performed for 50 epochs for consistency across experiments, while the model corresponding to the best validation performance observed within the 50-epoch training window was retained for evaluation.

### 5.2. Trade-Off Analysis

The second experiment is designed to identify the 2.5D U-Net model configuration that offers the best trade-off between segmentation performance and computational cost, under the practical constraint of real-time execution on a standard laptop.

**Figure 6:**
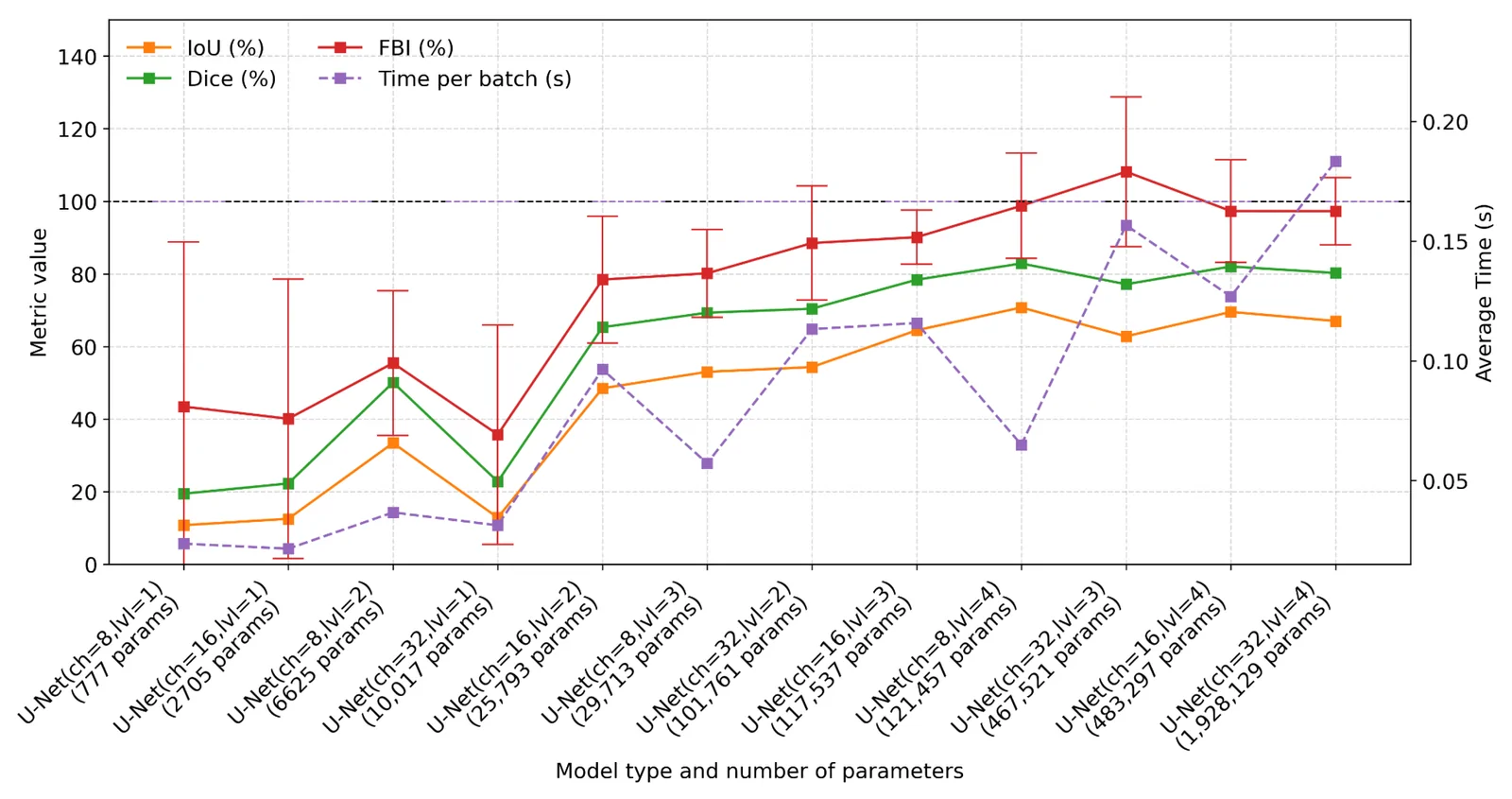
Segmentation performance (IoU, Dice, and FBI with standard deviation) and inference time per batch for different 2.5D U-Net architectures, evaluated with a fixed threshold of t=0.5. Each model is identified by its configuration (channels, levels) and the corresponding number of parameters. FBI values are displayed with error bars to indicate variability across the test dataset. Inference time is plotted using a secondary y-axis.

Figure 6 shows the performance of the tested 2.5D U-Nets with fixed threshold t=0.5 and lh=2, while varying the number of base channels ch ∈{8,16,32} and the number of downsampling levels lvl ∈{1,2,3,4}. The performance is measured through the classical IoU, Dice, and FBI metrics (see Section 4.2), as well as average inference time per batch (right vertical axis), measured under real-time execution conditions. A single horizontal reference line guides interpretation by marking the target FBI value of 100% (indicating ideal coverage of the GME region) when read against the left axis, while simultaneously denoting the maximum acceptable inference time per batch when read against the right axis, beyond which real-time responsiveness may be compromised.

All metrics are computed over the entire test set. Since accurate estimation of the overall GME burden is the primary objective of the proposed monitoring framework, FBI was considered the most clinically relevant calibration metric during model selection, while IoU and Dice were used to evaluate spatial segmentation consistency. For this reason, we include error bars around the FBI values, computed as FBI $\pm \;\sigma_{10}$, where $\sigma_{10}$ is the standard deviation calculated by first grouping the test dataset into batches of size 10 images and then computing the FBI over each batch.

In Figure 6, as expected, we observe that increasing the number of parameters generally leads to better performance, but higher inference times per batch. Thus, models with more than 100 k parameters begin to achieve higher Dice and FBI values. However, beyond approximately 500 k parameters, the performance gains tend to plateau. Meanwhile, the inference time per batch increases notably with model size, potentially introducing delays in real-time applications, especially on resource-constrained devices.

Since the real-time application aims to estimate the area of GME regions, we prioritize models with low FBI variability and a mean FBI possibly near 100%. FBI values above 100% were preferred to FBI below 100% as discussed in Section 4.2, overestimating the GME area is preferable to underestimating it, as missing relevant regions may have more serious consequences. Based on this criterion, the most promising models are U-Net(ch=8,lvl=3,4), U-Net(ch=16,lvl=3), and U-Net(ch=32,lvl=4), while all four tend to slightly underestimate the GME area, probably due to the dominance of the background, the last configuration exceeds hardware constraints, making it less suitable for deployment.

A final operating parameter is the segmentation threshold *t*, which is applied to the predicted probability map after network inference. Lowering *t* increases the number of pixels classified as GME, thereby generally increasing both TP and FP. Unlike the architectural parameters, *t* is not learned during training and can be modified without retraining the network or changing its computational complexity. Based on the preliminary experiments, we fixed t=0.4 as a common operating point for the quantitative evaluation. The same threshold was subsequently applied to all compared architectures and kept fixed across all LOPO folds.

**Figure 7:**
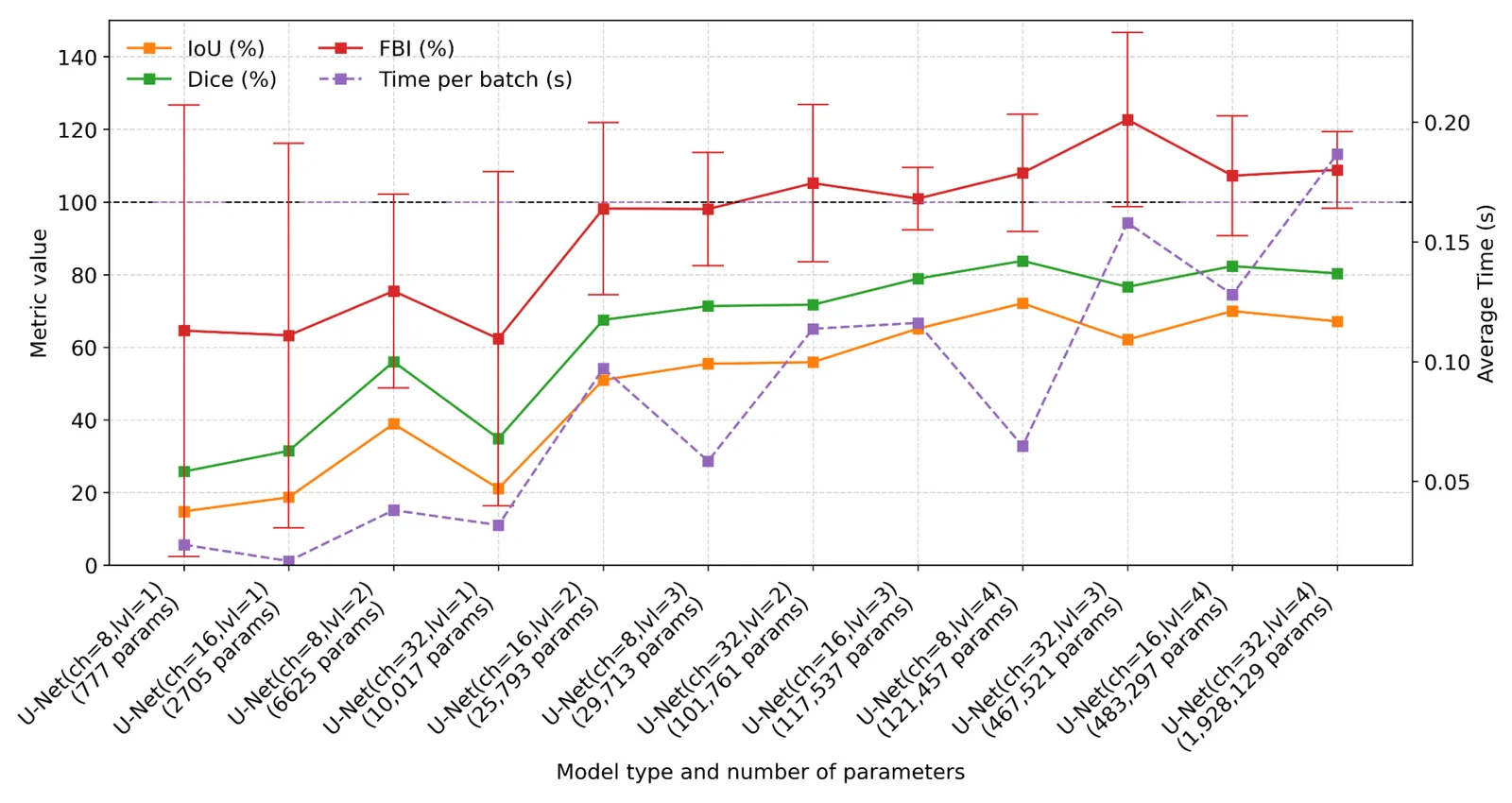
Segmentation performance (IoU, Dice, and FBI with standard deviation) and inference time per batch for different 2.5D U-Net architectures, evaluated with a threshold of t=0.4. Each model is identified by its configuration and number of parameters.

In Figure 7 we note that the U-Net(ch=8,lvl=4) and U-Net(ch=16,lvl=3) models exhibit low FBI standard deviation (i.e., high reliability), moderate network size (i.e., faster inference), and strong overall performance. Both are therefore suitable for real-time applications. However, we focus on the latter, as its slightly lower FBI variance makes it the more reliable choice, while IoU and Dice (i.e., spatial agreement) remain comparable between the two models.

This adjustment confirms that the selected model offers the best trade-off between performance, reliability, parameter count, and real-time feasibility.

### 5.3. Model Performance Evaluation

In this section, we present a comprehensive evaluation of the selected 2.5D U-Net model U(ch=16, lvl=3, lh=2, t=0.4), demonstrating its effectiveness on real data.

After selecting the final architecture through the trade-off analysis described above, we further evaluated the selected model using a leave-one-patient-out protocol. The selected architecture and operating threshold were fixed before the LOPO evaluation and were kept identical across all folds. Each LOPO model was initialized and trained from scratch using only the corresponding training patients. Because the architecture and threshold were selected on the same cohort, the LOPO results should be interpreted as within-cohort feasibility estimates rather than fully independent estimates of external generalizability. This evaluation was designed to assess patient-level generalization within the available cohort and to compare the proposed lightweight 2.5D U-Net with additional state-of-the-art segmentation architectures, namely Attention U-Net (AttUNet) [17], which introduces attention gates to focus the segmentation process on relevant image regions, and U-Net++ [18], which employs nested dense skip connections to improve feature fusion between encoder and decoder levels. The strict pixel-wise results of this comparison are reported in Table 1. Because the clinical objective is bubble detection and embolic-load estimation rather than exact contour coincidence at uncertain boundaries, we also report in Table 2 the radius-tolerant grace-zone evaluation defined in Section 4.2.

Table 1 shows that all evaluated architectures achieve comparable strict pixel-wise performance, but that generalization remains limited under the current low-patient regime. This suggests that the main limiting factor is the limited number of independent clinical acquisitions rather than the specific architectural choice. Within this setting, the proposed 2.5D U-Net remains close to the larger 2.5D variants of AttUNet and U-Net++, while still outperforming the 2D baselines despite using substantially fewer trainable parameters.

Table 2 provides the complementary grace-zone evaluation, in which small localization offsets around the bubble boundary are tolerated. As expected, the overlap-based scores increase markedly for all models under this relaxed criterion, while the overall ranking remains essentially unchanged: the 2.5D models still perform best, Attention U-Net yields the highest tolerant overlap scores, and the proposed 2.5D U-Net remains close behind despite its much smaller size. This behavior indicates that a substantial part of the residual error in Table 1 is due to small boundary mismatches rather than complete failures to detect bubbles.

**Figure 8:**
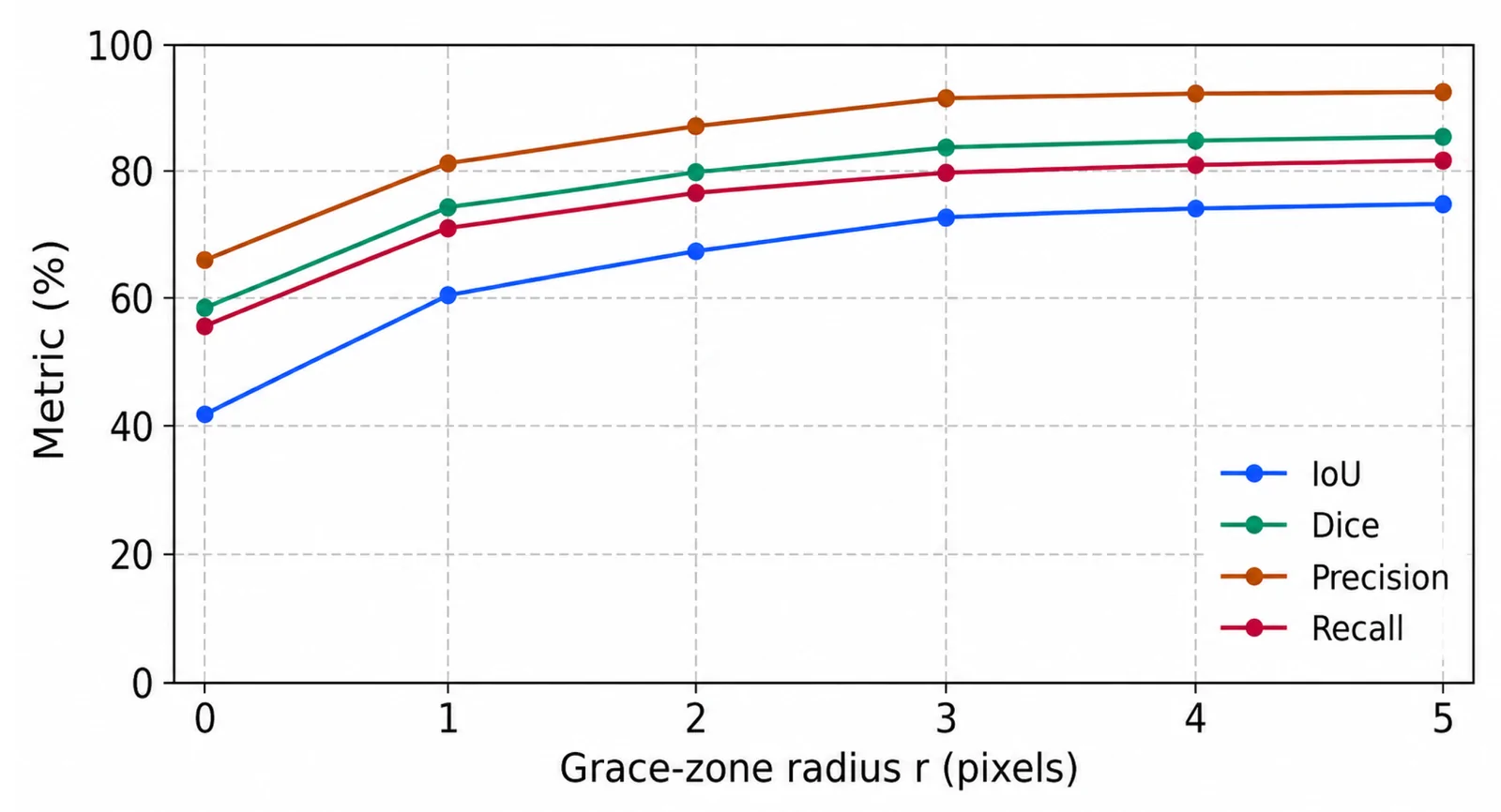
Sensitivity of the grace-zone radius *r* for the selected 2.5D U-Net.

To assess the dependence of the radius-tolerant evaluation on the choice of *r*, we additionally performed a sensitivity analysis for the selected 2.5D U-Net using r=0,…,5 pixels, where r=0 corresponds to the strict pixel-wise evaluation. The resulting mean IoU, Dice, precision, and recall values are shown in Figure 8. The largest increase is observed when moving from the strict evaluation (r=0) to a tolerance of only one pixel (r=1). For larger radii, the metrics continue to improve more gradually and tend to stabilize from approximately r=3 onward.

The sensitivity analysis also provides an empirical context for the r=3 value used in the main radius-tolerant evaluation. Rather than representing a uniquely optimal or clinically defined threshold, r=3 lies in the region where the metric curves begin to plateau, providing a representative small image-space tolerance while avoiding substantially larger relaxation of the spatial criterion. Importantly, the same qualitative conclusion is already evident at smaller radii and therefore does not depend critically on the specific choice of r=3. Because the physical pixel size varies across patients according to acquisition settings and zoom level, a fixed radius in pixels does not correspond to the same physical distance in every examination. The three-pixel radius should therefore be interpreted only as an image-space tolerance for complementary offline evaluation and not as a universal physical distance or clinical safety margin.

The substantial difference between the strict and radius-tolerant results provides information about the spatial distribution of the segmentation errors. In particular, it suggests that the models frequently localize the correct GME regions, while a considerable proportion of the residual pixel-wise error is concentrated near their boundaries. The principal limitation therefore appears to concern precise contour delineation rather than complete failure to localize the embolic regions. Accordingly, the grace-zone results characterize the nature of the errors and complement the strict pixel-wise metrics.

We also note that additional tests, not reported in this paper, indicated that reducing the number of parameters in the 2D setting did not appear to improve the results. Importantly, while the difference in parameter count is substantial, the observed differences in both the strict and grace-zone evaluations remain relatively small. This finding is particularly relevant for the intended clinical use case, where real-time execution, low latency, and lightweight deployment are essential constraints. Therefore, although generalization remains limited with the present dataset, the proposed architecture represents a practical step toward real-time emboli detection and quantitative GME burden estimation. Larger multi-patient datasets will be required to fully assess generalization and to determine whether more complex architectures can provide consistent performance gains.

### 5.4. Evaluation on GME-Negative Dataset

To complement the leave-one-patient-out evaluation with an external negative-control experiment, we analyzed the publicly available SR-TEE dataset, which contains transesophageal echocardiographic sequences without visible GMEs [16]. The dataset comprises 4394 frames from real TEE cases and 15,825 synthetically generated TEE frames, for a total of 20,219 frames. These data were completely unseen during model development and include cardiac views acquired or generated at different probe positions and zoom levels from those represented in our training dataset.

This experiment was designed as a negative-control test to determine whether the model produces spurious detections when applied to ordinary cardiac structures and echocardiographic background patterns from an independent source. Since no visible GMEs are present in these sequences, we assumed a completely black ground-truth mask for every frame. As a consequence, every pixel classified as GME was considered a false-positive pixel.

**Figure 9:**
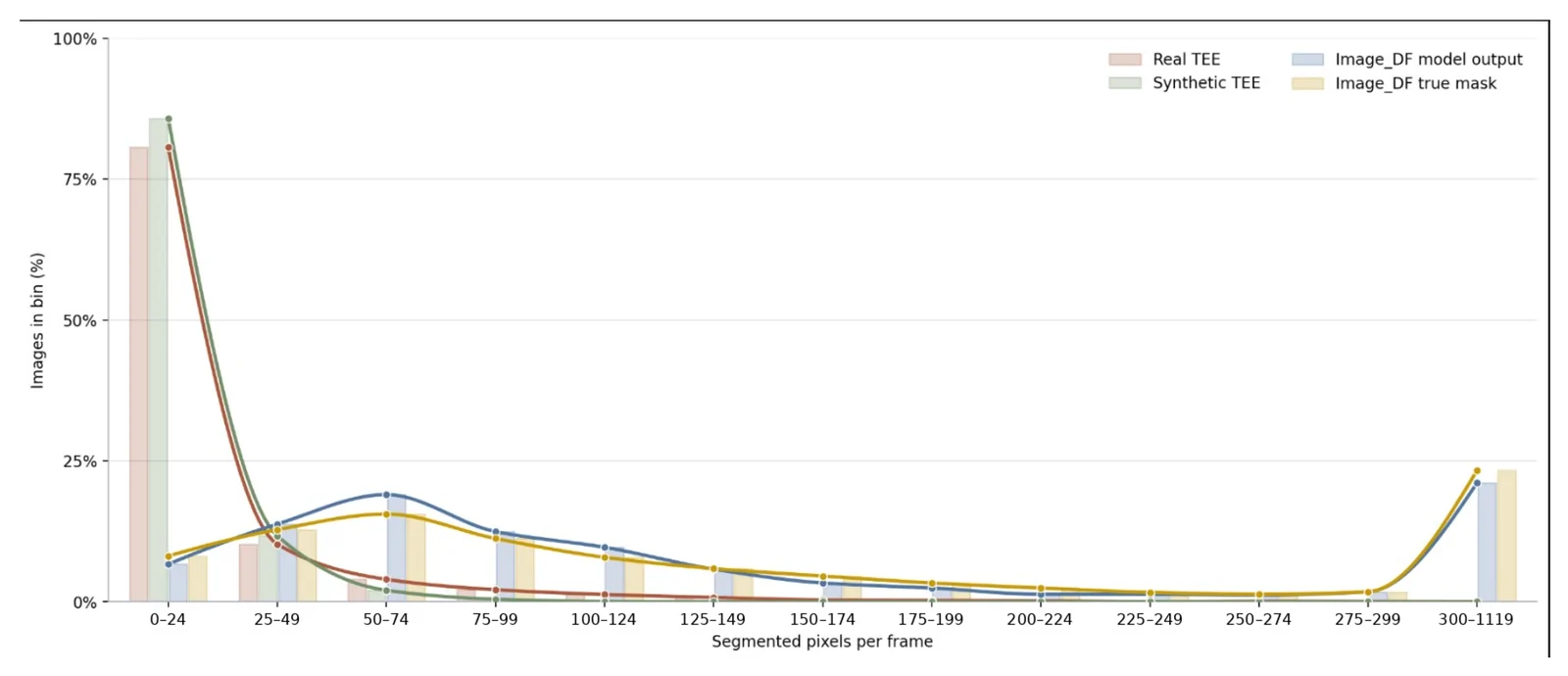
Distributions of the number of pixels classified as GME on the GME-positive dataset (blue), its corresponding ground-truth masks (yellow), and the predictions on the real (red) and synthetic (green) subsets of the GME-negative SR-TEE dataset.

To contextualize the negative-control results, we compared these outputs with those obtained on a GME-positive reference subset of the original dataset summarized in Table 3. This reference subset includes only frames whose ground-truth masks contain at least one GME-positive pixel. Figure 9 therefore compares four frame-wise distributions: the model predictions and corresponding ground-truth masks on this GME-positive subset, and the model predictions on the real and synthetic subsets of the GME-negative SR-TEE dataset. The distributions obtained on both GME-negative subsets are strongly concentrated near zero and are clearly separated from those observed on the GME-positive recordings. In particular, more than 90% of the predictions on the GME-negative data contain fewer than 50 false-positive pixels, an area approximately comparable to that of two average-sized manually segmented GMEs. Moreover, the model produced completely empty masks, containing no false-positive pixels, for 50% of the real TEE frames and 29% of the synthetic TEE frames. The median false-positive area was also only 5 pixels on the synthetic TEE subset, indicating that at least half of these frames contained no more than five false-positive pixels. This value is markedly lower than the medians observed on the GME-positive dataset, which were 95 pixels for the model outputs and 105 pixels for the ground-truth masks, i.e., approximately 100 pixels in both cases. More generally, both the predicted and ground-truth distributions on our GME-positive dataset are shifted toward substantially larger values, showing that the network produces a markedly stronger response when GMEs are present. This separation is also reflected in the maximum pixel counts: spurious predictions reached at most 315 pixels on the real TEE subset and 125 pixels on the synthetic subset. In contrast, the GME-positive distributions exhibit substantially heavier upper tails, with pixel counts approaching or exceeding 1000 pixels. These results indicate that the model does not systematically misclassify normal cardiac anatomy or echocardiographic background patterns as GME. Nevertheless, this negative-control experiment should not be interpreted as a substitute for validation on an independent GME-positive cohort, but as a complementary assessment of model specificity in the absence of visible emboli.

### 5.5. Real-Time Segmentation

Beyond the offline quantitative evaluation, the selected 2.5D U-Net was integrated into a real-time inference pipeline to assess its suitability for intraoperative use. This setup was designed not only to provide immediate visual feedback on the presence and spatial distribution of GMEs, but also to estimate their visible area during the intervention. The prototype was physically implemented and tested as an external real-time processing system connected to the Philips Epiq echocardiography unit through an HDTV USB 3.0 video-capture device.

**Figure 10:**
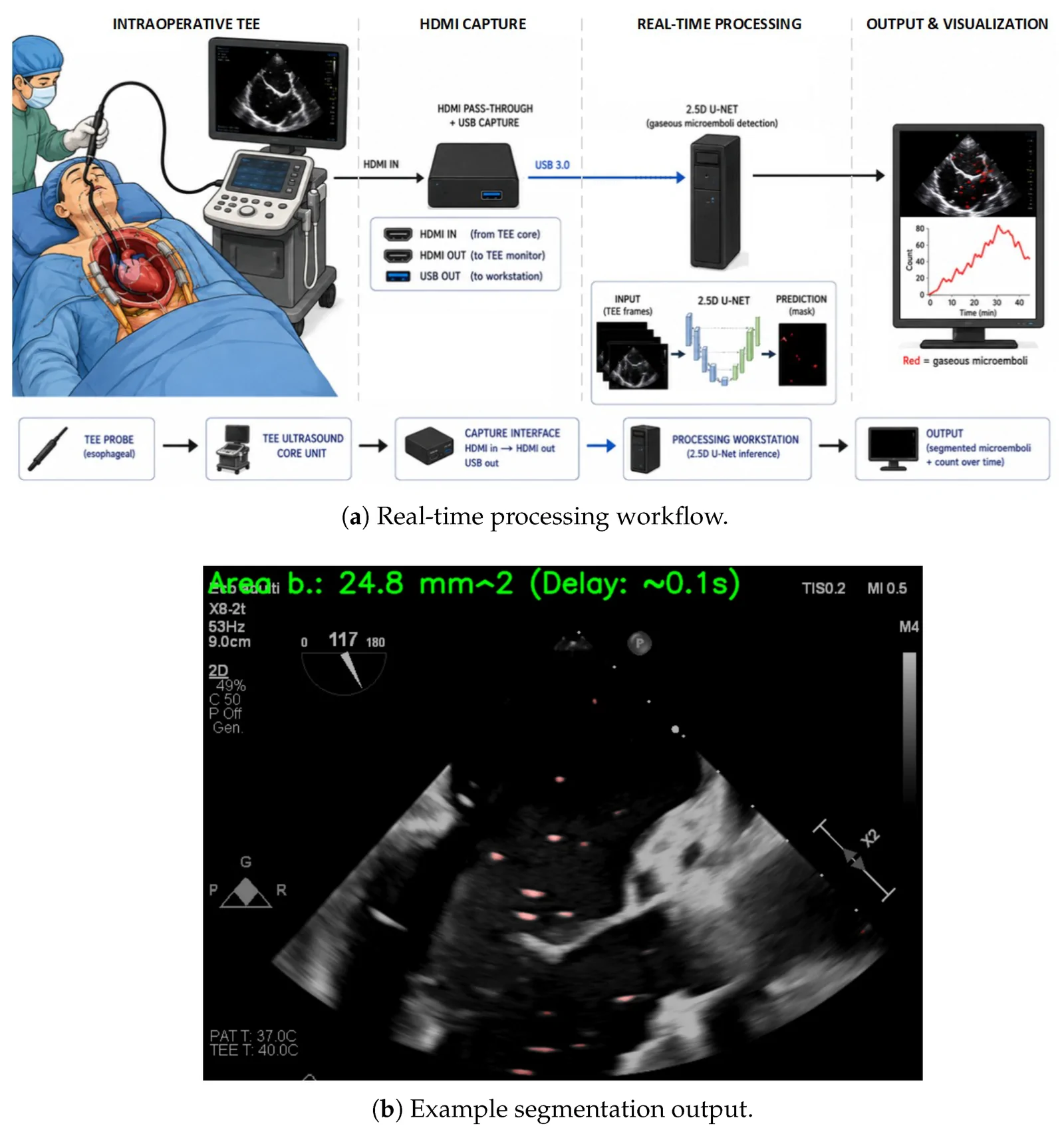
Real-time intraoperative GME monitoring framework. (**a**) Schematic representation of the complete processing pipeline: the intraoperative TEE video stream is mirrored through the capture interface, transferred via USB 3.0 to a local processing workstation, analyzed by the 2.5D U-Net, and returned as a real-time visualization of the detected GME regions and their quantitative evolution. (**b**) Representative frame acquired during real-time operation. Red overlays indicate GMEs detected by the selected 2.5D U-Net model U(ch=16, lvl=3, lh=2, t=0.4), while the estimated GME area is displayed at the top of the image.

Figure 10a summarizes the complete intraoperative processing workflow. The TEE video signal is mirrored through the capture interface so that the conventional echocardiographic visualization remains available to the clinical team, while the same stream is transferred to a local workstation for 2.5D U-Net inference. The resulting segmentation is then displayed as an additional real-time output without modifying the standard TEE acquisition workflow. Figure 10b shows a representative output generated in real time by the implemented system on a single frame captured during surgery.

At the top of the output screen, the GME area (Area b.) value is displayed, representing the estimated overall GME area. This estimate is computed by counting the number of pixels classified as positive (ones) in the network’s output mask, corresponding to the numerator of the FBI quantity. The pixel count is then rescaled to a physical measurement in ${\mathrm{mm}}^{2}$, taking into account the zoom level selected by the doctor operating the echocardiography system. The segmented area is computed as the product of the number of segmented pixels and the known physical area represented by a single pixel under the current acquisition settings. This provides a real-time quantitative assessment of the area visible during the intervention.

For visualization purposes, given that the video runs at 60 frames per second, we apply an exponential moving average (EMA), with $\alpha =\frac{1}{2}$, and update the GME area estimate every ten frames to ensure a readable, accurate, and interpretable value. In addition, Figure 10 reports an estimate of the processing delay, which becomes relevant when the available hardware does not fully support real-time performance. Specifically, this value is computed from the number of frames currently waiting in the processing queue and their corresponding acquisition time. Therefore, values below 0.2 s indicate that the system is operating, without accumulating additional delay due to inference or frame acquisition. Displaying this delay helps verify correct pipeline operation and allows the surgical team to assess whether latency remains within acceptable bounds. This measurement can be used during the procedure as a real-time indicator of GME presence and extent, and its reliability can be assessed directly by comparing the predicted GMEs with the live visual feedback. The quantitative characterization of GME may also be relevant beyond their total area. Experimental and numerical studies have shown that both the size and number of GMEs can influence infarction burden, with smaller gaseous microemboli being associated with a greater total volume of cerebral infarctions for a given air volume [19,20]. These findings highlight that individual GME size may provide complementary information to the total embolic-area estimate produced by the proposed framework. Moreover, in the postoperative phase, the network can be applied to provide the same quantitative information almost instantaneously, transforming the original data into a more interpretable format that may also be accessible to non-clinical personnel.

## 6. Conclusions

This work identified a lightweight 2.5D U-Net architecture as a practical and computationally efficient approach for real-time detection and quantification of gaseous microemboli in intraoperative cardiac ultrasound. By leveraging short-term temporal context, the proposed model improves the discrimination between moving emboli and static cardiac background structures while preserving low-latency execution compatible with intraoperative deployment.

A leave-one-patient-out comparison against Attention U-Net and U-Net++ further showed that all evaluated architectures experience limited generalization under the current low-data regime. This suggests that the primary limitation is the reduced number of independent clinical acquisitions rather than the specific architecture itself. Nevertheless, the proposed 2.5D U-Net achieved segmentation performance comparable to substantially larger models while requiring significantly fewer trainable parameters, which is particularly relevant for lightweight real-time clinical applications. U-Net-based architectures have shown strong adaptability across medical imaging tasks [21]. Although our study was conducted on a limited number of subjects using a single imaging device, the negligible predicted GME burden on the independent GME-negative dataset provides preliminary evidence that the proposed model does not systematically generate spurious detections in the absence of visible emboli. Whether the proposed protocol generalizes to other centers with similar acquisition settings remains to be evaluated on independent GME-positive cohorts.

Overall, the framework represents a step toward real-time AI-assisted monitoring of embolic load, with potential benefits for intraoperative decision-making and patient safety.

## Figures and Tables

**Table 1:** Leave-one-patient-out segmentation performance (threshold t=0.4). The dagger (^†^) denotes the proposed model.

Model	Params	IoU	Dice	AJI	Prec.	Rec.	FBI	ROC AUC
AttUNet 2.5D (ch=16,lvl=3)	494k	43.72 ± 10.21	60.24 ± 9.69	44.09 ± 11.23	61.10 ± 8.77	61.63 ± 15.65	104.74 ± 27.44	99.47 ± 0.51
U-Net++ 2.5D (ch=16,lvl=3)	11.5M	42.85 ± 12.24	59.10 ± 11.95	43.19± 13.28	65.22 ± 12.28	58.84 ± 18.81	97.62 ± 37.29	99.63 ± 0.36
U-Net 2.5D (ch=16,lvl=3) ^†^	118k	41.74 ± 12.11	57.98 ± 12.28	42.35 ± 13.75	65.12 ± 10.77	56.41 ± 18.50	92.91 ± 35.55	99.59 ± 0.42
U-Net++ 2D (ch=32,lvl=4)	13.9M	40.42 ± 12.44	56.62 ± 12.26	40.85 ± 13.26	67.76 ± 12.12	50.19 ± 14.67	78.84 ± 21.25	99.38 ± 0.63
AttUNet 2D (ch=32,lvl=4)	7.94M	40.54 ± 13.95	56.42 ± 14.65	40.95 ± 13.81	62.56 ± 7.65	56.88 ± 22.92	95.54 ± 39.86	99.41 ± 0.47
AttUNet 2D (ch=32,lvl=1)	103k	33.30 ± 14.79	48.36 ± 16.61	34.85 ± 13.99	58.94 ± 9.22	45.49 ± 23.47	77.66 ± 40.10	99.56 ± 0.40

**Table 2:** Leave-one-patient-out segmentation performance (threshold t=0.4) under the radius-tolerant grace-zone criterion (r=3 pixels). The dagger (^†^) denotes the proposed model.

Model	Params	IoU	Dice	Prec.	Rec.
AttUNet 2.5D (ch=16,lvl=3)	494k	77.58 ± 14.09	86.73 ± 9.31	92.44 ± 6.85	82.66 ± 11.18
U-Net++ 2.5D (ch=16,lvl=3)	11.50M	76.92 ± 17.63	85.90 ± 12.12	93.77 ± 6.14	82.47 ± 15.86
U-Net 2.5D (ch=16,lvl=3) ^†^	118k	73.95 ± 15.69	84.13± 11.34	92.55 ± 4.55	80.54 ± 15.72
U-Net++ 2D (ch=32,lvl=4)	13.89M	70.24 ± 17.93	81.33 ± 12.98	92.19 ± 12.91	75.24 ± 13.83
AttUNet 2D (ch=32,lvl=4)	7.94M	71.06 ± 21.56	81.28 ± 16.44	93.32 ± 4.60	75.70 ± 19.41
AttUNet 2D (ch=32,lvl=1)	103k	64.88 ± 24.30	78.60± 18.17	90.85± 9.72	74.13 ± 20.59

**Table 3:** Dataset summary and split information used for final model performance evaluation.

Variable	Value
Number of echocardiographic videos	8
Number of patients/procedures	8
Approximate duration per video	2 s
Frame rate	60 frames/s
Physical area per pixel (mean ± SD ${\mathrm{mm}}^{2}$/pixel)	0.0886±0.031
Frame resolution	600 × 800 pixels
Patch size after subdivision	300 × 400 pixels
Approximate number of patches	∼4000
Split strategy	Leave-one-patient-out cross-validation

## Data Availability

The data presented in this study are not publicly available due to privacy and confidentiality restrictions.
